# A dental workforce strategy to make Australian public dental services more efficient

**DOI:** 10.1186/s12960-019-0370-8

**Published:** 2019-05-30

**Authors:** Tan Minh Nguyen, Utsana Tonmukayakul, Hanny Calache

**Affiliations:** 1 Deakin University, 75 Pigdons Road, Waurn Ponds, Victoria 3216 Australia; 2 University of Melbourne, Parkville, Australia; 3 Peninsula Health, Frankston, Australia; 4 Coburg Hill Oral Care, Hill, Coburg, Australia; 5 La Trobe University, Bendigo, Australia; 6 North Richmond Community Health, North Richmond, Australia

**Keywords:** Child health, Oral health, Public/private, Health economics, Health policy, Health sector reform, Health systems research, Health workers, National health service, Public policy

## Abstract

**Background:**

Dental services can be provided by the oral health therapy (OHT) workforce and dentists. This study aims to quantify the potential cost-savings of increased utilisation of the OHT workforce in providing dental services for children under the Child Dental Benefits Schedule (CDBS). The CDBS is an Australian federal government initiative to increase dental care access for children aged 2–17 years.

**Methods:**

Dental services billed under the CDBS for the 2013–2014 financial year were used. Two OHT-to-dentist workforce mix ratios were tested: Model A National Workforce (1:4) and Model B Victorian Workforce (2:3). The 30% average salary difference between the two professions in the public sector was used to adjust the CDBS fee schedule for each type of service. The current 29% utilisation rate of the CDBS and the government target of 80% were modelled.

**Results:**

The estimated cost-savings under the current CDBS utilisation rate was AUD 26.5M and AUD 61.7M, for Models A and B, respectively. For the government target CDBS utilisation rate, AUD 73.2M for Model A and AUD 170.2M for Model B could be saved.

**Conclusion:**

An increased utilisation of the OHT workforce to provide dental services under the CDBS would save costs on public dental service funding. The potential cost-savings can be reinvested in other dental initiatives such as outreach school-based dental check programmes or resource allocation to eliminate adult dental waiting lists in the public sector.

## Key messages

- The costs to fund dental care under a universal healthcare system are expensive.
- Current Australian dental workforce models, which predominately rely on dentists to provide dental care, are inefficient to provide public dental services.
- Countries that are considering to embed dental services via universal healthcare systems should maximise the role of oral health therapists to provide more efficient public dental services.

## Introduction

Oral diseases remain one of the most prevalent non-communicable chronic diseases that affect 90% of the world’s population. Common oral diseases such as dental caries and periodontal disease share common risk factors with systemic diseases [1]. In Australia, oral conditions are the second most common cause of acute potentially preventable hospitalisations (PPH). Dental caries is the 10th most common cause of non-fatal burden of disease in Australia, totalling 71 889 years lived with disability [2]. In the State of Victoria, Australia, about 64% of oral conditions related to acute PPH were directly attributed to dental caries [3].

In 2015–2016, there were 67 266 PPH admissions for oral conditions [4]. PPH is defined as ‘admissions that are potentially avoidable through timely and accessible, primary healthcare’. Globally, this measure is considered to be a ‘high-level’ health system performance indicator [5]. In 2016, the average cost of hospital in-patient episode of care for dental extractions and restorations was AUD 3041 [6]. Nationally, AUD 205M could potentially be saved if PPH admissions due to oral conditions could be averted [4]. The Australian health expenditure in 2014–2015 on oral health reached AUD 9.6B. Only 23.8% of this expenditure was contributed by government funding [7].

The goal to establish universal access to public dental care is often the topic of government debates [8]. Australian public dental services have traditionally been the responsibility of the state/territory governments. Two federal dental programmes, the Chronic Disease Dental Scheme (CDDS; 2007–2012) and the Medicare Teen Dental Plan (MTDP; 2008–2013), were previously introduced and implemented to address population inequalities on access to dental services. Evaluations of these programmes, however, have shown that (1) the CDDS was not cost-effective [9] and poorly utilised in rural and remote areas [10] and (2) the MTDP had low utilisation rates (highest rate recorded at 29%) by eligible teenagers [11, 12] despite most claims have no out-of-pocket expenses. Reasons for low utilisation of both programmes, particularly in the rural and remote areas where the inequality is more prominent, remain unknown.

The focus on dental services for children has since expanded to include children aged 2–17 years. This new scheme was branded the Child Dental Benefits Schedule (CDBS) in 2014 [13]. Under the CDBS, eligible children can claim up to AUD 1000 of dental benefits over 2 years. The CDBS included a wide range of dental treatment services such as restorations (fillings), removal of teeth and root canal treatment, which was not included in the MTDP. Two reviews of the CDBS share similar concerns to the MTDP, that is, regarding low utilisation rates [13, 14]. The current rate at 29% falls short of the government target of 80% [13]. There is evidence that the Australian healthcare system is not achieving optimal oral health outcomes for children aged 0–12 years [15]. Currently, 29% of children aged 5–6 have never visited a dental practitioner, and 26% of dental caries remain untreated in that population group [16].

The dental workforce in Australia consists of a range of dental practitioners that include dentists, dental specialists, dental hygienists (DH), dental therapists (DT), oral health therapists (OHT) and dental prosthetists. Dentists and dental specialists provide comprehensive dental services under the definition of dentistry. The scope of practice of dentists and dental specialists covers complex dental procedures such as root canal treatment, surgical removal of teeth and fabricating fixed dental prosthesis (dental implants, cast crowns and dental bridge work). DH, DT and OHT, which make up the oral health therapy workforce, have a more narrow scope of practice and are focused on prevention. Their scope of practice is limited to routine dental examinations, preventive procedures, the placement of non-complex restorations (fillings) and non-surgical periodontal treatment (removal of plaque and calculus from teeth).

In general, dentists and the oral health therapy workforce manage the two most common oral diseases, dental caries and periodontal disease, at various levels of complexity. The length of training for dentists is between 5 and 7 years (5-year bachelor degree or 3-year bachelor degree combined with a 4-year postgraduate degree) compared to 3-year bachelor degree for OHT. OHT have combined skillsets of DH and DT. Traditionally, DH and DT qualifications were either a 2-year certificate or a 2-year diploma. However, DT training programmes no longer exist in Australia due to the emergence of training dual-qualified OHT [17, 18].

Dental services provided to children have been the historical legacy of the DT role in addressing children’s unmet dental needs [19]. The New Zealand model for utilising DT in school-based services started in 1921 and has rapidly spread to 54 other countries including Australia [20]. Several government reports identified the importance to better utilise the OHT workforce to their full scope of practice [21–23]. One possible reason for low utilisation rates of the MTDP and CDBS dental programmes may be due to an existing inefficient dental workforce model. In this paper, DH, DT and OHT are collectively referred to as the OHT workforce unless otherwise explicitly stated.

Under the current workforce skill mix ratio, there is a reliance on the ‘over-qualified’ dentist workforce to provide less complex dental services. Nationally, the dental workforce comprises of 21% of OHT and 79% for dentists [24], an OHT-to-dentist workforce skill mix ratio of 1:4. In the dental public sector in Victoria, dentists account for 61% of the dental workforce, an OHT-to-dentist workforce skill mix ratio of 2:3. Dentists, the most expensive member of the dental team [22], have been the primary providers for the CDDS, MTDP and CDBS. Therefore, this study aims to quantify the potential cost-savings of a hypothetical increased utilisation of the OHT workforce for providing dental services via the CDBS dental programme.

## Methods

De-identifiable data used in the present study are publicly available. Therefore, ethics approval was not required. This research was performed according to principles from the Declaration of Helsinki.

### Data source

Data on dental services billed under the CDBS for the 2014–2015 year was retrieved electronically and publicly available [25]. However, the data does not provide information as to who provided the service. Dental providers were identified by using two OHT-to-dentist workforce skill mix ratios and applying a salary difference between the two dental professions to adjust the total CDBS claims for each ratio.

### Salary difference

The average salary difference between OHT and dentist employed in the public sector was used from eight state and territory jurisdictions (Table 1). The ‘on-cost’ of employment was not used because it is proportionally different and would not affect the average salary difference. The estimated salary difference is 30%, which means the OHT workforce would earn 30% less than dentists for dental services within their scope of practice (Table 2 and Appendix).

**Table 1:** The 2013/2014 salary differential between OHT and dentists employed in the public sector, by state and territory in Australia

State/territory	Salary difference (%)
Australian Capital Territory [57]	15.6
New South Wales [58, 59]	29.8
Northern Territory [60, 61]	20.0
Queensland [62]	35.6
South Australia [63]	41.0
Tasmania [64, 65]	48.3
Victoria [66, 67]	16.0
Western Australia [68, 69]	33.8
Mean salary differential	30.0

**Table 2:** Summary of dental treatment services that can be provided by the dental practitioner divisions

Service type	DH	DT	OHT	Dentist
Diagnostics	✓	✓	✓	✓
Preventive	✓	✓	✓	✓
Periodontics	✓	✘	✓	✓
Oral surgery	✘	88311 and 88316 only	88311 and 88316 only	✓
Endodontics	✘	88411 and 88414 only	88411 and 88414 only	✓
Restorative services	✘	✓	✓	✓
Prosthodontics	✘	✘	✘	✓
General services	88911 only	88911 only	88911 only	✓

88311—extraction of tooth (first tooth extracted on day)

88316—additional extraction of tooth

88411—direct pulp capping

88414—pulpotomy

88911—palliative care

*DH* dental hygienist, *DT* dental therapist, *OHT* oral health therapist

### Workforce models

Two OHT-to-dentist workforce skill mix ratios were considered. For Model A National Workforce, the cost-savings was estimated using the Australian dental workforce skill mix ratio of 1:4 [24]. For Model B Victorian Workforce, the Victorian public sector dental workforce skill mix ratio of 2:3 was applied [22]. The proportion of each type of dental service provided was assigned against the type of dental provider by percentage (Table 3). This modelling approach was adopted in previous work [26].

**Table 3:** Dental service provision was weighted for Model A National and Model B Victoria. The dental workforce ratio for Model A and Model B is approximately 1:4 and 2:3, respectively

Models of workforce distribution	OHT	Dentist
Model A National (1:4)	19.6%	80.4%
Model B Victoria (2:3)	39%	61%

*OHT* oral health therapist

### Scenario analysis and discounting

The cost effects were modelled according to the current CDBS utilisation rate of 29%. A one-way sensitivity analysis was performed for the government target of 80% CDBS utilisation rate. All costs were calculated in 2014 Australian dollars. A discount rate did not apply since costs are consumed within 1 year. Data analysis was performed using Excel 2016 (Microsoft Corporation).

### Assumptions

The following assumptions were applied in this analysis:

- The type and number of dental services provided to children 2–17 years are evenly distributed across the state and territory jurisdictions.
- Dental services were weighted according to each type of provider by workforce size percentage.
- All the children receiving dental treatment received at least a comprehensive oral examination (item code 88011).

## Results

The total CDBS expenditure in the 2014/2015 year was AUD 537M. A summary of the cost allocation according to dental service type and age groups is shown in Table 4. The total costs for the 0–4, 5–14 and 15–17 age groups were AUD 18.8M, AUD 376.5M and AUD 141.8M, respectively. The projected total costs under Model A National Workforce and Model B Victorian Workforce are AUD 511M and AUD 475M resulting in the potential cost-savings of AUD 26.5M and AUD 61.7M, respectively. For the one-way sensitivity analysis calculated using a 2.7-fold increase in dental service utilisation, the potential cost-savings would be AUD 73.2M and AUD 170.2M, respectively. A summary of the cost allocation for each type of provider is shown in Table 5.

**Table 4:** The total cost for Medicare benefits claims under the CDBS by dental service type in the 2014–2015 financial year

Service type	0–4 age group (A$)	5–14 age group (A$)	15–17 age group (A$)	Subtotal (A$)
Diagnostics	5 244 036	50 479 640	18 764 669	74 488 345
Preventive	4 348 506	121 296 380	44 984 411	170 629 297
Periodontics	11 740	246 037	240 960	498 738
Oral surgery	430 620	39 108 772	8 789 368	48 328 760
Endodontics	125 322	4 551 556	3 583 657	8 260 535
Restorative services	8 503 913	158 133 500	64 770 100	231 407 513
Prosthodontics	3 220	379 504	309 428	692 152
General services	168 618	2 292 021	329 127	2 789 766
Total	18 835 975	376 487 410	141 771 720	537 095 105

*DH* dental hygienist, *DT* dental therapist, *OHT* oral health therapist

**Table 5:** The estimated costs for dental services provided under the CDBS against the type of dental service for Models A and B

Type of dental service	Model A (National 1:4)				Model B (Victoria 2:3)		
	DH (A$)	DT/OHT (A$)	Dentist (A$)	Subtotal (A$)	OHT (A$)	Dentist (A$)	Subtotal (A$)
Diagnostics	3 411 108	6 784 314	59 861 769	70 057 191	20 249 553	45 437 891	65 687 444
Preventive	7 785 212	15 483 921	137 124 425	160 393 559	46 215 798	104 083 871	150 299 669
Periodontics	22 756	45 258	400 805	468 819	135 086	304 230	439 316
Oral surgery	0	4 172 480	42 320 870	46 493 349	11 635 674	31 574 730	43 210 404
Endodontics	0	223 659	7 938 492	8 162 151	623 710	7 362 463	7 986 174
Restorative services	0	22 474 406	199 046 958	221 521 364	62 673 728	141 164 565	203 838 293
Prosthodontics	0	0	692 152	692 152	0	692 152	692 152
General services	7 011	13 943	2 759 595	2 780 549	41 617	2 729 842	2 771 459
Total	11 226 086	49 197 981	450 145 066	510 569 133	141 575 166	333 349 744	474 924 910

*DH* dental hygienist, *DT* dental therapist, *OHT* oral health therapist

## Discussion

This study estimated the potential economic benefits for utilising the OHT workforce from the Australian healthcare system perspective for dental services under the CDBS as a case study. The oral health workforce profile has changed since the landmark 1993 Nuffield Foundation report recommended the establishment of the ‘oral health therapist’, to complement existing dental services mainly provided by dentists [27]. It is widely recognised that the OHT workforce provides high-quality and cost-effective dental services within their scope of practice, which enables dentists to focus on more complex procedures [28, 29].

Oral health workforce modelling in the United Kingdom shows there is a significant demand for OHT in optimising the dental workforce skill mix cost-effectively [30–32]. For example, only 30% of dentists would be required, and the number of DT would need to increase tenfold to achieve 52% in salary cost-savings [32]. Positive associations were identified for increasing productivity among dental practices that employ DH in the US private sector [33, 34]. More recently, there is a growing demand for training DT in the United States of America to meet unmet community needs for children, low-income families and rural communities [19, 35–37].

The range of dental services provided by the OHT workforce is diverse [17, 18] which could result in various levels of economic benefits for the community if the OHT workforce plays a critical role in primary healthcare. An independent policy report on public dental funding noted that changing the oral health workforce favouring OHT over dentists would reduce the cost of subsequent phases of a universal dental scheme [38]. The State of Victoria is one example where the dental workforce is more cost-effectively utilised than the national workforce skill mix due to greater utilisation of the OHT workforce.

This paper quantified the potential cost-savings if public funded dental services for children reflected the 30% salary difference between the OHT and dentist based on the CDBS fee schedule. From our modelling work, there are three proposals for consideration:

- Option 1: Status quo: the government will continue to pay benefits at dentists’ fee rates for dental services provided by OHT. The monetary surplus is retained as ‘profit’, which can be an incentive for not-for-profit public dental services to deliver more services.
- Option 2: Introduce a two-tier CDBS fee structure to reflect the 30% salary differential between OHT and dentists. In other words, dental services delivered by OHT are 30% cheaper than the same service provided by dentists. Under this option, it is necessary for OHT to obtain a Medicare provider number to bill for services directly. Currently, OHT can only use a dentists’ Medicare provider number. Eligible children and adolescents would be able to access more services under the AUD 1000 2-year capped allowance if their care is provided predominately by the OHT workforce.
- Option 3: Introduce an overall 30% reduction in CDBS fee structure: the cost-savings would be more significant than option 2. However, this option could create a disincentive for dentists to provide dental services under their scope of practice to the CDBS eligible population and potentially widen the gap of the inequity of access to public dental care among socioeconomic disadvantage population.

Although funding for dental treatment is an essential part of the healthcare system, there are alternative preventive models of care that are worth considering. For example, an outreach school-based dental check-up programme provided by the OHT workforce increased dental utilisation for Victorian public dental services for children from low-income families [15, 39, 40]. An economic evaluation determined that the intervention was less costly and more clinically effective than standard care [40]. Another strategy that could be adopted in Australia is enabling non-dental practitioners to provide oral health prevention services. Positive impacts have been demonstrated in both the Australian context [41, 42] and the United States of America [43–48]. Studies from the United Kingdom [49–51] and Sweden [52] currently capitalise on the expanded role of dental assistants to provide preventive services to children. This model is currently being explored in Victoria [22, 53].

Another potential resource allocation from the estimated cost-saving could be reinvestment to eliminate adult public dental waiting list, which can be up to 3 years [23]. Adult public dental waiting lists are a major problem in Australia dental care system since it is reliant on government funding. It is estimated that AUD 46.6M (AUD 50M–100M) is required to reduce the 2013 Australian public dental waiting list from 263 043 to zero; costs would increase to AUD 111.4M if dental services were contracted to the private sector [54]. The cost-saving based on Model A could be allocated to this, but only half of the required budget will be met, whereas the cost-saving from Model B will not only set the waiting list to zero but also provide a surplus of AUD 15.1M. There is also an additional economic benefit for utilising the OHT workforce since the model on costs required to manage the 2013 waiting only included the cost of dentists to provide adult dental services. International countries considering to fund or expand dental services under a universal healthcare system can make public dental services more affordable through the maximal utilisation of the OHT workforce.

Although the potential cost-savings are obvious, it remains unknown that greater utilisation of the OHT workforce would increase CDBS uptake. Observations from past government reviews on the MTDP and CDBS dental programmes suggest that consumer-driven demand is relatively low which means dental cost subsidisation may not improve access to dental care [12–14]. However, the supply of the dental profession and willingness to participate in federal dental schemes is also critical. Greater dentist participation in Medicaid for children’s dental care in the United States of America has been associated with dentist density and high reimbursement rates [55]. Naturally, if dentists are remunerated better by not participating in subsidised schemes, utilisation rates for federal dental schemes would be less than ideal. Hence, increasing the OHT workforce, a workforce that may have a greater willingness to participate would potentially boost the CDBS uptake rate. Unequivocally, the global literature review of utilising DT in public school-based programmes increases access to dental care for children compared to the US private practice dentist-led model [19, 56].

Furthermore, the goal to implement universal dental care goes beyond the fee structures for dental practitioners and an adequate level of government funding. It is unknown whether there is sufficient infrastructure to provide dental services through the public and private sector. Therefore, the costs to establish accessible dental clinics by consumers must be considered but is beyond the scope of this paper. These costs could be offset by having a paradigm shift in the way the federal government currently funds the number of tertiary education programmes for the OHT workforce and dentists. A typical postgraduate dental programme costs more than AUD 300 000. Proportionally, domestic OHT students account for 24% of the combined OHT and dental student enrolment [26].

Alternatively, other strategies could make public dental services more affordable under a universal healthcare system. Firstly, since OHT qualifications require less time for training. The Australian government could consider gradually reducing the number of students enrolled in dental programmes. The decreasing government-supported domestic dental student enrolment would result in a reduction in government expenditure on tertiary education. Secondly, to address an inefficient workforce skill mix ratio, a rapid increase in OHT numbers could be achieved by replacing dental student positions with OHT student positions. As a result, an increased overall supply of OHT in the workforce could facilitate a more affordable investment in establishing universal dental care compared to the status quo. The major assumptions discussed above qualitatively discuss some of the main limitations of our study. Therefore, the results should be interpreted with caution.

## Conclusion

In summary, the potential cost-savings from the publicly funded CDBS dental programme for children can be achieved through maximal utilisation of the OHT workforce from the Australian healthcare perspective. Policy-decision makers should consider the important role of the OHT workforce in achieving universal dental care. The potential cost-savings could be reinvested in other dental initiatives that would increase access to dental care.

## Appendix

**Table 6:** The service provision weights of individual dental services according to the dental practitioner division scope of practice for Models A and B

Item code	Service description	Model A National			Model B Victoria	
		DH	OHT/DT	Dentist	OHT	Dentist
88011	Comprehensive oral exam	0.0657	0.131	0.804	0.390	0.610
88012	Periodic oral examination	0.0657	0.131	0.804	0.390	0.610
88013	Oral examination—limited	0.0657	0.131	0.804	0.390	0.610
88022	Intraoral periapical or bitewing radiograph—per exposure	0.0657	0.131	0.804	0.390	0.610
88025	Intraoral radiograph—occlusal, maxillary, mandibular—per exposure	0.0657	0.131	0.804	0.390	0.610
88111	Removal of plaque and/or stain	0.0657	0.131	0.804	0.390	0.610
88114	Removal of calculus—first visit	0.0657	0.131	0.804	0.390	0.610
88115	Removal of calculus—subsequent visit	0.0657	0.131	0.804	0.390	0.610
88121	Topical application of remineralisation and/or cariostatic agents, one treatment	0.0657	0.131	0.804	0.390	0.610
88161	Fissure and/or tooth surface sealing—per tooth (first four services on a day)	0.0657	0.131	0.804	0.390	0.610
88162	Fissure and/or tooth surface sealing—per tooth (subsequent services)	0.0657	0.131	0.804	0.390	0.610
88213	Treatment of acute periodontal infection—per visit	0.0657	0.131	0.804	0.390	0.610
88221	Clinical periodontal analysis and recording	0.0657	0.131	0.804	0.390	0.610
88311	Removal of a tooth or part(s) thereof—first tooth extracted on a day	0	0.140	0.860	0.390	0.610
88314	Sectional removal of a tooth or part(s) thereof—first tooth extracted on a day	0	0	1	0	1
88316	Additional extraction requiring removal of a tooth or part(s) thereof, or sectional removal of a tooth	0	0.140	0.860	0.390	0.610
88322	Surgical removal of a tooth or tooth fragment not requiring removal of bone or tooth division—first tooth extracted on a day	0	0	1	0	1
88323	Surgical removal of a tooth or tooth fragment requiring removal of bone—first tooth extracted on a day	0	0	1	0	1
88324	Surgical removal of a tooth or tooth fragment requiring both removal of bone and tooth division—first tooth extracted on a day	0	0	1	0	1
88326	Additional extraction requiring surgical removal of a tooth or tooth fragment	0	0	1	0	1
88351	Repair of skin and subcutaneous tissue or mucous membrane	0	0	1	0	1
88384	Repositioning of displaced tooth/teeth—per tooth	0	0	1	0	1
88386	Splinting of displaced tooth/teeth—per tooth	0	0	1	0	1
88387	Replantation and splinting of a tooth	0	0	1	0	1
88392	Drainage of abscess	0	0	1	0	1
88411	Direct pulp capping	0	0.140	0.860	0.390	0.610
88412	Incomplete endodontic therapy (tooth not suitable for further treatment)	0	0	1	0	1
88414	Pulpotomy	0	0.140	0.860	0.390	0.610
88415	Complete chemo-mechanical preparation of root canal—one canal	0	0	1	0	1
88416	Complete chemo-mechanical preparation of root canal—each additional canal	0	0	1	0	1
88417	Root canal obturation—one canal	0	0	1	0	1
88418	Root canal obturation—each additional canal	0	0	1	0	1
88419	Extirpation of pulp or debridement of root canal(s)—emergency or palliative	0	0	1	0	1
88421	Resorbable root canal filling—primary tooth	0	0	1	0	1
88455	Additional visit for irrigation and/or dressing of the root canal system—per tooth	0	0	1	0	1
88458	Interim therapeutic root filling—per tooth	0	0	1	0	1
88511	Metallic restoration—one surface—direct	0	0.140	0.860	0.390	0.610
88512	Metallic restoration—two surfaces—direct	0	0.140	0.860	0.390	0.610
88513	Metallic restoration—three surfaces—direct	0	0.140	0.860	0.390	0.610
88514	Metallic restoration—four surfaces—direct	0	0.140	0.860	0.390	0.610
88515	Metallic restoration—five surfaces—direct	0	0.140	0.860	0.390	0.610
88521	Adhesive restoration—one surface—anterior tooth—direct	0	0.140	0.860	0.390	0.610
88522	Adhesive restoration—two surfaces—anterior tooth—direct	0	0.140	0.860	0.390	0.610
88523	Adhesive restoration—three surfaces—anterior tooth—direct	0	0.140	0.860	0.390	0.610
88524	Adhesive restoration—four surfaces—anterior tooth—direct	0	0.140	0.860	0.390	0.610
88525	Adhesive restoration—five surfaces—anterior tooth—direct	0	0.140	0.860	0.390	0.610
88531	Adhesive restoration—one surface—posterior tooth—direct	0	0.140	0.860	0.390	0.610
88532	Adhesive restoration—two surfaces—posterior tooth—direct	0	0.140	0.860	0.390	0.610
88533	Adhesive restoration—three surfaces—posterior tooth—direct	0	0.140	0.860	0.390	0.610
88534	Adhesive restoration—four surfaces—posterior tooth—direct	0	0.140	0.860	0.390	0.610
88535	Adhesive restoration—five surfaces—posterior tooth—direct	0	0.140	0.860	0.390	0.610
88572	Provisional (intermediate/temporary) restoration—per tooth	0	0.140	0.860	0.390	0.610
88574	Metal band	0	0.140	0.860	0.390	0.610
88575	Pin retention—per pin	0	0.140	0.860	0.390	0.610
88576	Metallic crown—preformed	0	0.140	0.860	0.390	0.610
88579	Bonding of tooth fragment	0	0.140	0.860	0.390	0.610
88597	Post—direct	0	0	1	0	1
88721	Partial maxillary denture—resin, base only	0	0	1	0	1
88722	Partial mandibular denture—resin, base only	0	0	1	0	1
88731	Retainer—per tooth	0	0	1	0	1
88733	Tooth/teeth (partial denture)	0	0	1	0	1
88736	Immediate tooth replacement—per tooth	0	0	1	0	1
88741	Adjustment of a denture	0	0	1	0	1
88761	Reattaching pre-existing clasp to denture	0	0	1	0	1
88762	Replacing/adding clasp to denture—per clasp	0	0	1	0	1
88764	Repairing broken base of a partial denture	0	0	1	0	1
88765	Replacing/adding new tooth on denture—per tooth	0	0	1	0	1
88766	Reattaching existing tooth on denture—per tooth	0	0	1	0	1
88768	Adding tooth to partial denture to replace an extracted or decoronated tooth—per tooth	0	0	1	0	1
88776	Impression—dental appliance repair/modification	0	0	1	0	1
88911	Palliative care	0.0657	0.131	0.804	0.390	0.610
88942	Sedation—intravenous	0	0	1	0	1
88943	Sedation—inhalation	0	0	1	0	1

*DH* dental hygienist, *DT* dental therapist, *OHT* oral health therapist

## Abbreviations

CDBS: Child Dental Benefits Schedule
CDDS: Chronic Disease Dental Scheme
DH: Dental hygienist
DT: Dental therapist
MTDP: Medicare Teen Dental Plan
OHT: Oral health therapist
PPH: Potentially preventable hospitalisations
UK: United Kingdom
US: United States
